# Effects of a combination of herbal extracts (modified Ojayeonjonghwan (Wuzi Yanzong wan)) on partial urethral obstruction-induced detrusor overactivity in rats: impact on the nitric oxide pathway and oxidative stress

**DOI:** 10.1186/s12906-019-2467-y

**Published:** 2019-03-14

**Authors:** Sangrak Bae, Kyu Won Lee, Hyun Cheol Jeong, Bong Hee Park, Woong Jin Bae, Chang Hee Han, Sae Woong Kim

**Affiliations:** 1 Department of Urology, Uijeongbu St. Mary’s Hospital, College of Medicine, The Catholic University of Korea, Seoul, Republic of Korea; 2 Department of Urology, Seoul St. Mary’s Hospital, College of Medicine, The Catholic University of Korea, Seoul, Republic of Korea; 3 Kangdong Sacred Heart Hospital, Seoul, Republic of Korea

**Keywords:** MO formula, Detrusor overactivity, Urine, Overactive bladder, Alternative medicine

## Abstract

**Background:**

We investigated the effects of a berry mixture formula (modified Ojayeonjonghwan (Wuzi Yanzong Wan, MO formula) on detrusor overactivity (DO).

**Methods:**

The MO formula consisted of 5 seeds obtained from 5 types of berry plants. Twenty-four Sprague-Dawley rats were randomly assigned to four groups: sham-operated (control), partial urethral obstruction-induced DO (DO group), 0.03 mg/kg solifenacin-treated DO (solifenacin group) and 200 mg/kg MO formula -treated DO (berry mixture). The control and overactive groups were administered distilled water for 4 weeks, and the solifenacin and MO formula groups were treated with the respective medication for 4 weeks. After treatment, cystometrography was performed. At the endo of cystometrography, their bladder tissues were used for identifying the muscarinic receptors, endothelial nitric oxide synthase(eNOS), RhoA, Rock-I & II, 8-hydroxy-2′ –deoxyguanosine(8-OHdG), superoxide dismutase(SOD), interleukin-6 &-8(IL-6, IL-8), and tumor necrosis factor-alpha(TNF-a). The tissues were stained and the muscle-to-collagen ratio was identified.

**Results:**

The presence of the muscarinic receptors were not significantly different between the solifenacin and MO formula groups. However, significant differences were found between the solifenacin and MO formula groups in terms of eNOS, RhoA, Rock-I and -II levels. The muscle-to-collagen ratio was statistically lower in the DO and solifenacin groups; however, no significant difference was observed between the control and MO formula groups. Under oxidative stress, SOD showed a similar result as 8-OHgG. The MO formula group exhibited anti-inflammatory effects, showing that no significant difference was found between the control and MO formula groups regarding values of IL-6, IL-8, and TNF-a. However, the DO and solifenacin groups showed increased IL-6, IL-8, and TNF-a levels. Cystometrography showed that the OAB and solifenacin groups having a significantly lower value than the control and MO formula groups. The mean contraction interval was shorter in the DO, MO formula, and solifenacin groups and the highest in the control group.

**Conclusions:**

The MO formula exhibited a similar pharmacologic effect to that of solifenacin, with anti-inflammatory and antioxidant effects. Enhancement of the MO formula by the nitric oxide pathway affected DO including BPH-related DO. The MO formula may be one of the alternative choices of anticholinergics, a treatment for DO.

## Background

Overactive bladder (OAB) is an unpleasant urinary condition that decreases the quality of life of individuals. This condition is also known as a bladder storage/filling disorder and tends to increase with age. Frequent urination, urgency, urge-related incontinence, and nocturia are common symptoms associated with an OAB [1]. Anticholinergics such as solifenacin are widely used to manage the OAB syndrome. These agents act on the OAB by blocking the binding of acetylcholine to the muscarinic receptor on the detrusor muscle. This blockage inhibits the involuntary contraction of the urinary bladder. Patients who have received anticholinergics commonly complain about some of the drug-induced complications associated with intake. These include dry mouth, constipation, and increased residual urine in the urinary bladder. Treatment compliance is, therefore, lower and is positively related to longer treatment duration owing to some of the above-mentioned complications (dry mouth and constipation) [2]. Recently, newer drugs such as the b3 agonist have been associated with a decrease in the common complications of OAB. This anticholinergic drug selectively targets the urinary bladder and only a small proportion of patients complained about the unpleasant symptoms associated with intake [3]. However, these new drugs have not been observed for a long duration and their compliance or associated complications have yet to be proposed. The onabotulinum toxin injection is used for intractable DO, painful bladder syndrome, and other complications. These medications are also associated with many adverse events or unwanted side effects.

In males, benign prostatic hyperplasia (BPH) is a common aging-related condition, and long-standing BPH leads to detrusor overactivity (DO) or OAB. Following treatment to resolve bladder outlet obstruction (BOO), several patients experience unpleasant symptoms, especially that of bladder storage. In some cases, urinary tract infection or a change in the structure of the detrusor muscle occurs. BPH has also been shown to correlate with erectile dysfunction [4].

Alternative medicine is globally used and is associated with different names in different countries. In Japan, Kampo medicine is widely used in general hospitals, especially in the urology division [5]. Several Kampo formulas for frequent urination, nocturia, and sense of residual urine (e.g., Go-sha-Jin-Ki-Gan, Hachi-Mi-Ji-Ou-Gan, Go-Rin-San, et al) exist [5]. In Korea, ‘Dong UI Bo Gam: Principles and Practice of Eastern Medicine’ is a well-known oriental medicine book. In Dong UI Bo Gam, “Ojayeonjonghwan (Wuzi Yanzong Wan)” is composed of five types of berries: *Psyllium, Lycium chinense Miller*, *Rubus coreanus Miquel*, *Cuscuta chinensis Lam*, and *Schisandra chinensis Baillon*. The new berry mixture formula is a modification of Wuzi Yanzong Wan where *Psyllium* is replaced with *Cornus officinalis Sieb. et Zucc*. Several studies have shown that the modified Ojayeonjonghwan (MO) formula has anti-inflammatory, antioxidant [6], anti-apoptotic, and hypolipidemic effects, elicited through the nitric oxide pathway and another pathway via in vivo experiments [7]. Previous studies using the herbal formula (MO formula) have demonstrated increased levels of nitric oxide and cyclic GMP, and a similar effect was demonstrated with the phosphodiesterase type 5 inhibitor (PDE5I) [8]. Moreover, the MO formula has been used to exhibit antioxidant effects and anti-inflammatory effects on the bladder [6, 7, 9]. A previous study has also shown that PDE5I is related to lower urinary tract symptoms and affects the nitric oxide pathway [10–14].

We hypothesized that the improvement in the nitric oxide pathway by the MO formula is related to lower urinary tract symptom (LUTS), and further, lower instances of OAB or DO caused by bladder outlet obstruction (BOO). Thus, the aim of this study was to investigate the effects of a berry mixture, the MO formula, on partial urethral obstruction-induced DO.

## Methods

### MO formula

MO formula is currently being developed by the Kemimedi (KMD) Company (Seoul, republic of Korea) for the treatment of infertility. Originally, KMD Company, which develops oriental herbal medicines, developed this product as a health care supplement. It includes *Cornus officinalis Sieb. Et Zucc* (32%), *Lycium chinense* Mil (32%), *Rubus coreanus* Miquel (16%), *Rubus coreanus* Miquel (16%), and *Schisandra chinensis* Baillon (4%). MO formula was obtained from Andong Excellent Medicinal Herbs Distribution Center Co., Ltd. (Andong Korea). The formal identification of the plant material was undertook by S.Y. Hwang who was officer of R&D center of KEMIMEDI (KMD). Voucher specimens (KH204-CO, KH204-LC, KH204-RC, KH204-CC, and KH204-SC) of each plant were deposited at the R&D center of KEMIMEDI (KMD) Co. Ltd. (Andong, Korea). In the present study, 400 mg/kg MO formula was orally administered for 2 weeks. The MO formula was prepared as a mixture of the five herbs. 20 Kg of five herb products was extracted with 200 L of 30% distilled ethanol. After that, each products refluxed at 98 ± 2 °C for 3 h. After the herb extract was filtered and the liquid from the filtrate was removed using a rotary evaporator and a spray dryer.

### Features of the MO formula

The quality of the MO formula was confirmed using its marker compounds. Each of the components was selected as a marker compound as in another study [7]. The HPLC profile of the MO formula and its marker compounds were the same as that reported by Jang et al. [7].

### Animal groups and study design

A total of 24, 8-week-old, Sprague-Dawley female rats weighing 250–300 g were used. The rats were supplied by Orient Bio Inc. (Gyeonggi-do, Korea) and were treated using the protocol approved by the Institutional Animal Care and Use Committee at the School of Medicine, The Catholic University of Korea (Approval Number: CUMC- 2015-0175-01). Rats were also handled according to the guidelines set by the National Institutes of Health (NIH). The rats were fed a standard rat feed and were granted free access to food and water. The rats were also maintained in a 12 h light-dark cycle, a room temperature of 20 ± 2 °C and a relative humidity of 50 ± 10% throughout the experiment. The 24 female rats were randomly divided into four groups: 1) a sham operation only (no partial urethral occlusion, control group), 2) a partial urethral occlusion-induced DO (DO group), 3) a DO group treated with solifenacin, and 4) a DO group treated with 200 mg/kg of the MO formula. We used a rat model showing persistent DO induced by partial urethral obstruction [15]. All 24 rats were subjected to an abdominal incision to expose the urinary bladder and urethra. In the persistent, detrusor overactive group, and the DO groups treated with solifenacin and the MO formula, a 25 G angio-sheath was placed on top of the urethrovesical junction. We then ligated the urethra with 3–0 nylon to create a partial bladder outlet obstruction model followed by sheath removal. Both ends of the nylon were pulled into the vaginal cavity through the previously made incision. After 2 weeks, the partial urethral occlusion was released by cutting the previously placed nylon through the vagina. The control and overactive groups were administered distilled water for 4 weeks, and the solifenacin- and the MO formula-treated groups were treated with the respective medication for 4 weeks. To prevent urinary tract infection, cephazolin was injected subcutaneously at 7 mg/kg.

### Cystometrography

After the 4-week treatment period, a cystometrogram (CMG) was obtained of all rats. Subcutaneous 1.2 mg/kg urethane was injected as the anesthetic. Under anesthesia, midline laparotomy was performed to expose the bladder. A 25 G needle was then inserted into the bladder lumen, with a polyurethane tube connected to the needle. This tube was also connected to a pressure transducer to record the intravesical pressure as well as a Harvard syringe pump for saline infusion by a 3-way stopcock. The rate of saline infusion was 0.04 ml/min. The detrusor contraction, its interval and contraction pressure were recorded using a polygraph (Grass 7D, Grass Institute Co., Quincy, MA).

### Effect on oxidative stress

In the bladder wall, oxidative stress was evaluated based on Superoxide dismutase (SOD) and 8-OHdG levels. The SOD level was measured using a SOD Assay Kit-WST (Dojindo Laboratories, Kumamoto, Japan). In addition, the decrease in the rate of superoxide-mediated reduction of nitroblue tetrazolium was monitored at 450 nm using a spectrophotometer. The oxidatively modified DNA, 8-OHdG level, was measured to identify the level of DNA oxidative stress. Total DNA was extracted from the bladder tissue using a DNeasy Blood & Tissue Kit (Qiagen, Valencia, CA, USA). The 8-OHdG level was measured using a DNA oxidation kit (Highly Sensitive 8-OHdG Check ELISA; Japan Institute for the Control of Aging, Fukuroi, Japan). Absorbance was measured at 450 nm after final color development after the addition of 3, 3′, 5, 5′-tetramethylbenzidine. Tissue sample concentration was calculated from a standard curve and corrected for DNA concentration.

### Cytokine analysis for anti-inflammatory effect

To identify the anti-inflammatory effect of MO formula, proinflammatory cytokine such as tumor necrosis-α (TNF- α), and interleukin-8 and 4 (IL-8 and IL-4) were analyzed. The enzyme-linked immunosorbent assay (ELISA) was used as the analysis tool. After checking the cystometrogram, blood was obtained from rats following sacrificing. Blood centrifugation was then performed at 3000 rpm, at 4^°^*C* for 10 mins, followed by immediate transfer of the supernatant to a tube. The concentration of each cytokine was measured every 5 mins, for a total of 30 mins. The immunoassay ELISA kit (R&D systems, Minneapolis, MN, USA) and the spectrophotometer at 450 nm were used according to the manufacturers’ protocols.

### Western blot

After cystometrography, rats were euthanized by exsanguination method and the bladder tissues were obtained and frozen in liquid nitrogen. The frozen bladder tissue was grounded to a fine powder with a mortar and pestle, and cooled in liquid nitrogen. The total protein in the bladder was extracted using a cell lysis buffer (20 mM Tris-HCl pH 7.5, 150 mM NaCl, 1 mM NaEDTA, 1 mM EDTA, 1% Triton, 2.5 mM sodium pyrophosphate, 1 mM β-glycerophosphate, 1 mM Na3VO4, 1 μg/mL leupeptin, and 1 mM phenylmethylsulfonyl fluoride). Protein extracts were quantified using the BCA Protein Assay reagent (Thermo Scientific, Rockford, IL, USA) and the quantified proteins (60 μg) were boiled in a loading buffer (62.6 Mm Tris-HCl pH 6.8, 2% sodium dodecyl sulfate [SDS], 0.01% bromophenol blue, 10% glycerol, and 100 mM DTT). The proteins were then loaded onto a lane of a 4 to 12% SDS-polyacrylamide gel for electrophoretic separation. Proteins were transferred onto Hybond-ECL nitrocellulose membranes (Amersham Biosciences, Freiburg, Germany), and equal protein loading was verified by Ponceau-S staining (Sigma Aldrich Co., St. Louis, MO, USA). The membranes were blocked by treatment with 5% non-fat milk in tris-buffered saline containing 0.1% Tween 20. The membranes were also probed with endothelial NOS (1:1000, BD Pharmingen, San Diego, CA, USA), Rho A (1:1000, BD Pharmingen, San Diego, CA, USA), ROCK-I (1:1000, BD Pharmingen), ROCK-II (1:1000, BD Pharmingen), M2 (1:1000; Abcam, Cambridge, MA, USA), M3 (1:500; Abcam, Cambridge, MA, USA), and β-actin, followed by incubation with the corresponding secondary antibodies (Santa Cruz Biotechnology, CA, USA) conjugated with horseradish peroxidase. The densitometric analysis of band intensity was performed using the Luminescent Image Analysis System (LAS-3000; Fujifilm, Tokyo, Japan).

### Histological analysis

After CMG completion, the bladder wall was excised after the rats were euthanized, and divided into two sections. These two individual sections were used to conduct and identify the histological evaluation and oxidative stress, respectively. One bladder section was fixed in 20% buffered formalin for 96 h and the other section was frozen at − 80 °C. The bladder tissue was embedded in paraffin, cut into 4 mm transverse sections and stained with Hematoxylin-Eosin to identify the collagen-to-muscle ratio.

### Statistical analysis

Statistical analysis was performed using SPSS 18.0 (SPSS Inc., Chicago, IL, USA). The data of each group was compared using the one-way ANOVA test. Significance was defined as a *P* value < 0.05.

## Results

### Cystometrography

**Fig. 1:**
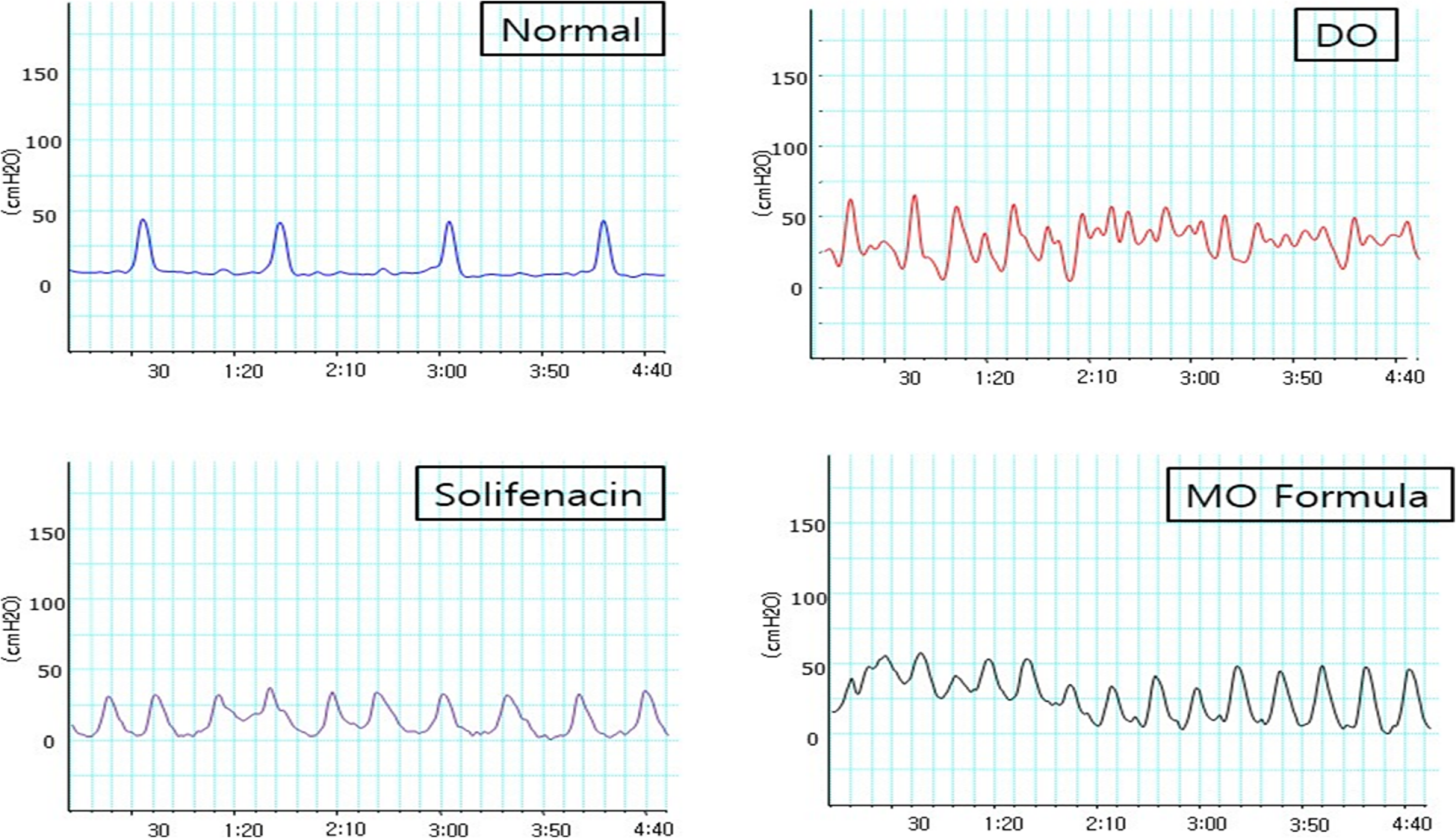
CMG on each group. Normal (sham operation group) and for making detrusor overactivity model, After 2 weeks of partial urethral obstruction, detrusor overactive model was identified in cystometrogram

**Fig. 2:**
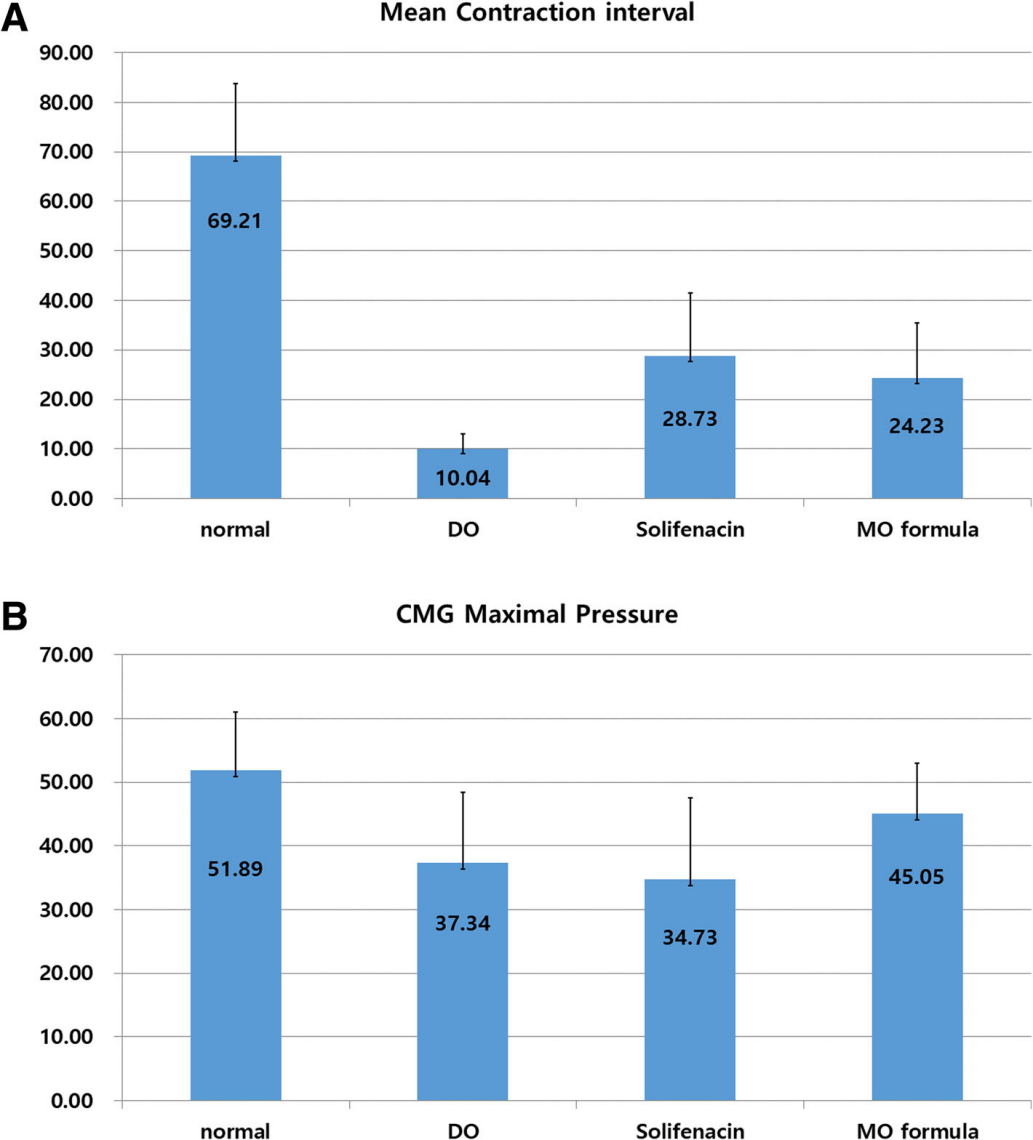
Contraction feature of each group. **a** Mean contraction interval. Compared to normal group, DO, solifenacin, and MO formula show significant short contraction interval, but there is no statistical difference between solifenacin and MO formula. **b** Mean maximal pressure on CMG. In maximal pressure, the highest pressure in the normal group and the lowest pressure in the solifenacin group

On the CMG, the BOO in the partial urethral occlusion groups (persistent DO group, solifenacin group, MO formula group) showed a low maximal detrusor contraction pressure and short contraction intervals (Fig. 1). The DO, solifenacin, and MO formula groups had a significantly short contraction interval compared to that of the control group (*P* < 0.001). The solifenacin and MO formula groups had no significant difference when compared to each other (*p* = 0.899). The control group had the highest maximal contraction pressure on CMG, whereas the solifenacin group had the lowest. A significant difference was observed between the control group and the solifenacin group in terms of maximal contraction pressure on CMG; however, the other groups showed no significant differences when compared to each other (*p* = 0.039) (Fig. 2b).

### Muscarinic receptor expression

**Fig. 3:**
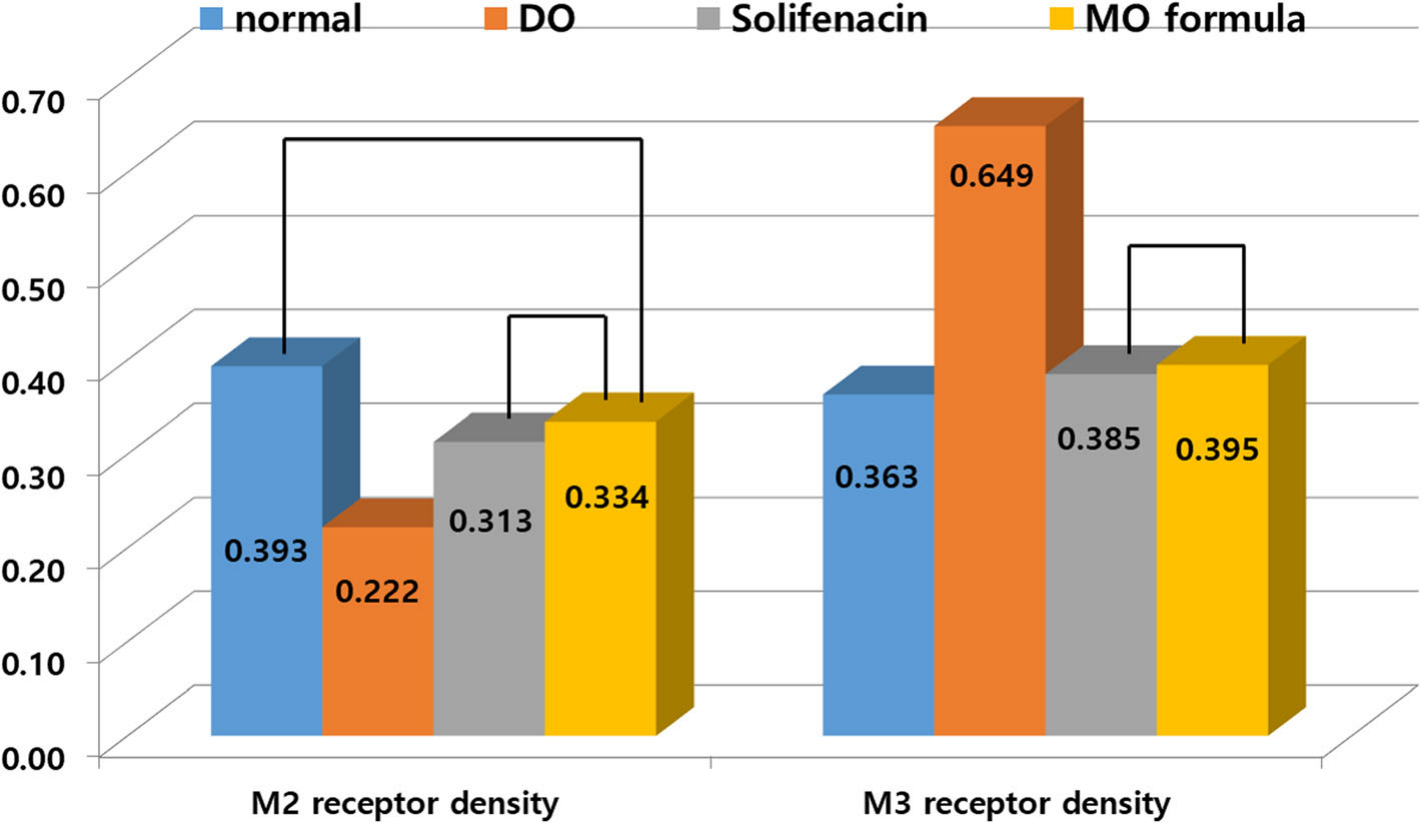
Expression of muscarinic receptor density. The DO group showed the lowest M2 receptor density and the highest M3 recpetor density, showing statistically significant differences. However, there was no significant difference between solifenacin and MO formula group

The DO group had a significant increase in M3 receptor density and the lowest M2 receptor density when compared to the observations in control group (*p* < 0.001, *p* < 0.001, respectively, Fig. 3). The solifenacin and MO formula groups showed higher M3 receptor and lower M2 receptor expression than the control group. There were no significant differences between the solifenacin and MO formula groups in terms of M3 and M2 density (*p* = 0.988, *p* = 0.854, Fig. 3). The MO formula group and the control group had no significant differences in M2 receptor density (*p* = 0.171).

### Nitric oxide related change

**Fig. 4:**
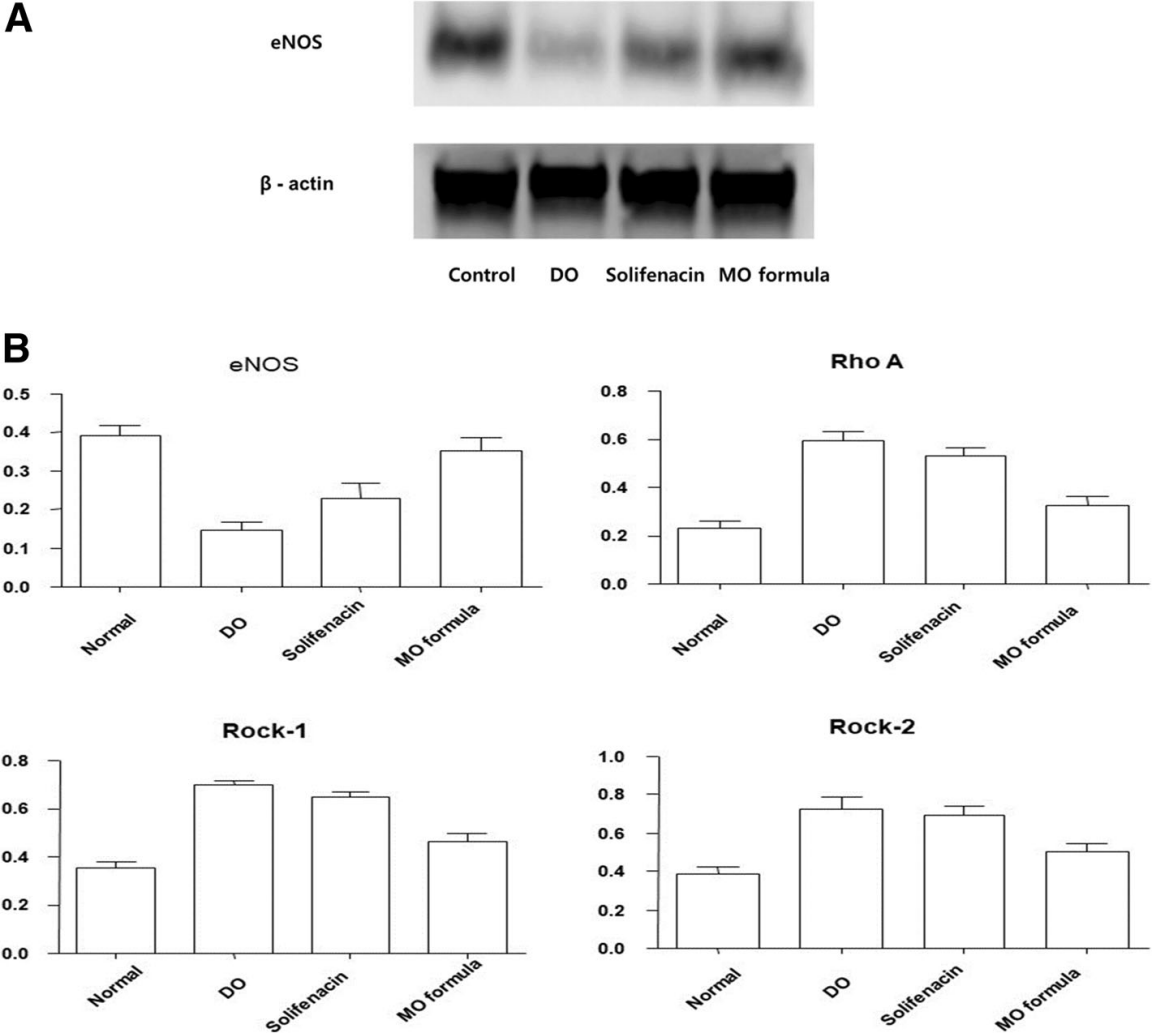
Nitric oxide related change. **a** Western blot analysis. **b** Difference between each group in eNOS, Rho, Rock-1, Rock-2

Compared to that of the control group, the DO group had the lowest level of endothelial nitric oxide synthase. The MO formula group had a similar extent of eNOS expression as the control group (Fig. 4a). The levels of Rho A and Rock-I and -II were the highest in the DO group and the lowest in the control group, respectively (Fig. 4b). Rho A showed a significant difference between the control group and the DO and solifenacin groups (*p* < 0.01). However, no significant difference was observed between the control group and the MO formula group (*p* = 0.307). For Rock-I, the DO, solifenacin, and MO formula groups had significant differences when compared to the control group (*p* = 0.00, *p* = 0.00, and *p* = 0.037, respectively). Rock-2 showed a significant difference in the control and MO formula groups when compared to the DO group. There was no significant difference between the DO and solifenacin groups (*p* = 0.937), and between the solifenacin and MO formula groups (*p* = 0.066).

### Oxidative stress

**Fig. 5:**
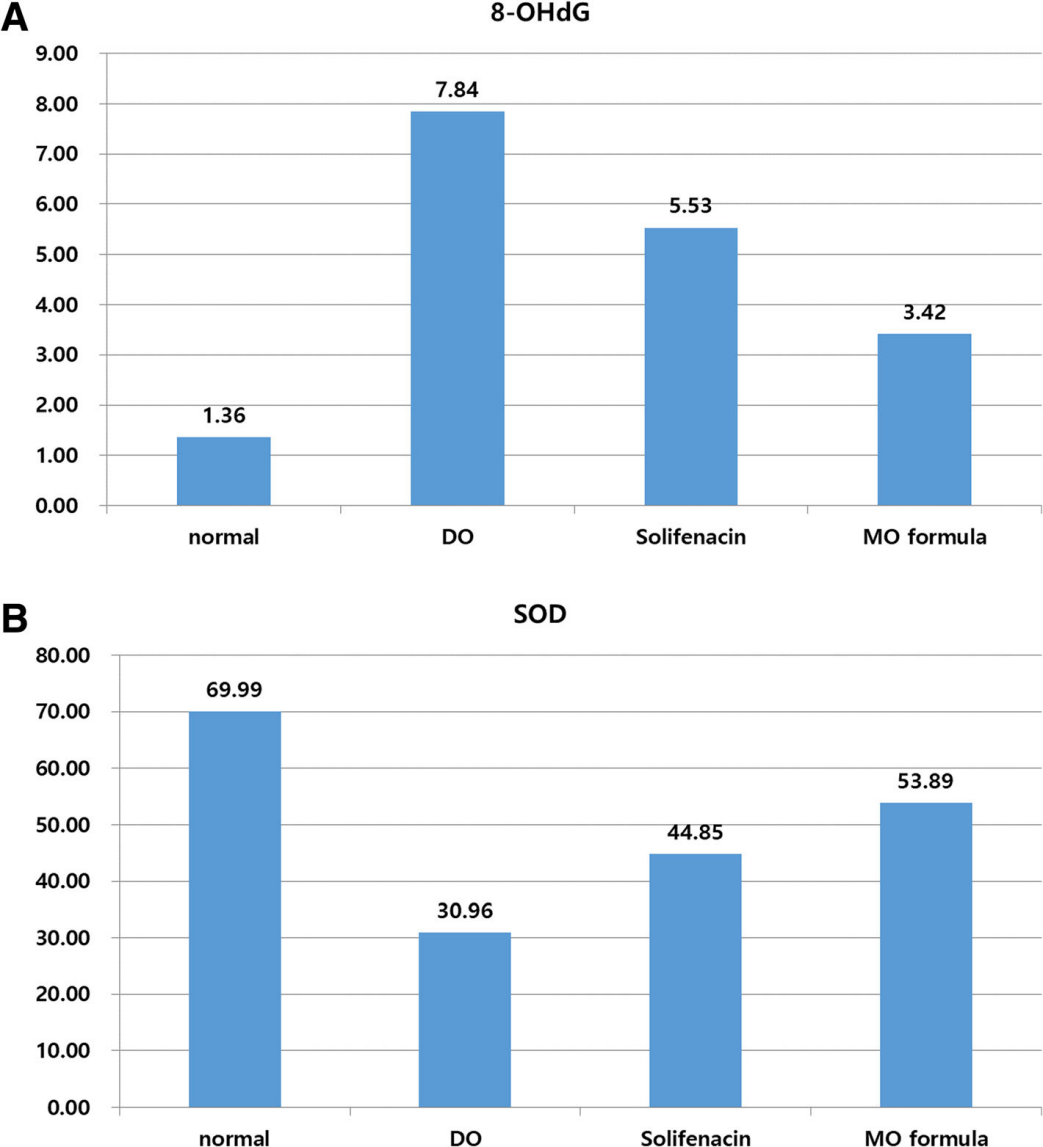
Parameter for oxidative stress defense. **a** 8-OHdG. **b** SOD

One of the parameter of oxidative stress, the 8-OHdG level was the highest in the DO group, whereas the lowest level was observed in the control group. However, in the inter-group analysis, the control group showed a difference when compared to the DO/solifenacin group, whereas no difference was observed when compared to the MO formula group. No significant difference was observed between the solifenacin group and the MO formula group (*p* = 0.139) (Fig. 5).

In the SOD results, the control group was observed to have the highest level of SOD, whereas the DO group had the lowest level. Based on the inter-group analysis, a similar statistical feature was observed for 8-OHdG (Fig. 5). It was confirmed that the DO group was the most vulnerable to oxidative stress with the oxidative stress being the most and the protective effect being the lowest. However, the MO formula group had more oxidative stress than the control group, and the protective effect was lower than that of the control group. However, it was not statistically different from the control group, unlike the solifenacin or DO group.

### Anti-inflammatory effect

**Fig. 6:**
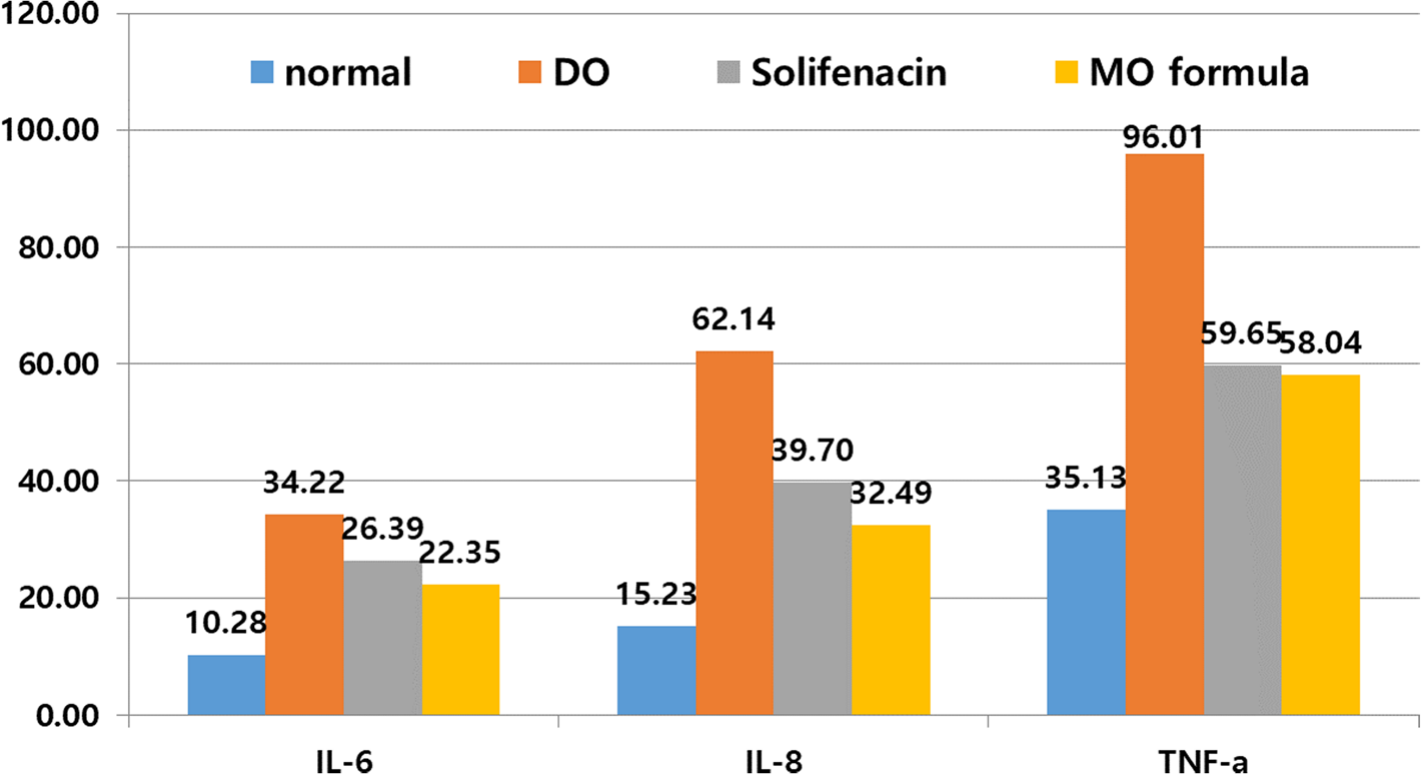
proinflammatory cystokine analysis

The production of the inflammatory cytokines including interleukin-6 (IL-6), interleukin-8 (IL-8), and tumor necrosis factor alpha (TNF-a) is shown in Fig. 6. The three inflammatory cytokines showed a high increased level in the DO group. The MO formula group had a significant difference in IL-6 and TNF-a compared to the control group (*p* = 0.028, *p* = 0.025); no difference was observed between the MO formula group and the control group in IL-8, *p* = 0.081. Compared to the solifenacin group, the MO formula group did not show any significant differences in the cytokines (*p* = 0.718, *p* = 0.701, *p* = 0.996). Compared with the control group, the expression of inflammatory cytokines was increased, while the expression of inflammatory cytokines was less than that of the solifenacin group. IL-8 was found to have an anti-inflammatory effect against inflammatory changes, which may be secondary effects of partial urethral obstruction, such that there is no statistical difference compared to the control group (Fig. 6).

### Histological analysis

**Fig. 7:**
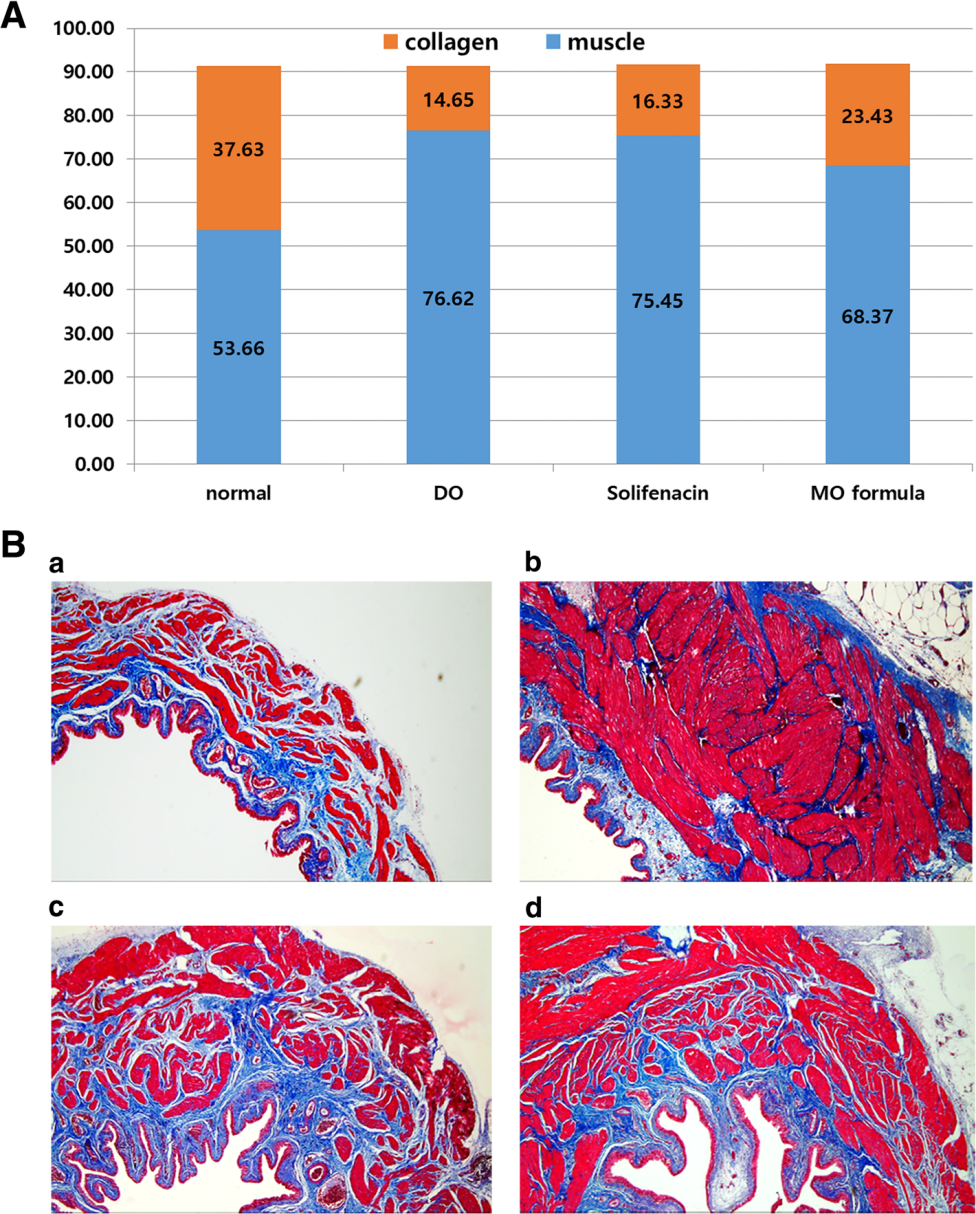
Microscopic Changes in Rat Bladder. **a** Muscle-to-Collagen ratio. **b** Histology on Masson-Trichrome stain. Control. DO. Solifenacin. MO Formula

For the histological examination, Masson trichrome staining was performed. On staining, the collagen tissue was colored green or blue, and the muscle was colored red. When the muscle-to-collagen ratio was compared, the control group was observed to have the lowest muscle-to-collagen ratio (1: 0.7), whereas the least muscle hypertrophy was observed in the DO group (1: 0.19). In the solifenacin group, muscle hypertrophy was remarkably similar to that in the DO group (1:0.22). In the MO formula group, increased muscle hypertrophy was observed compared to that of the control group; however, muscle hypertrophy was slightly lesser in the control group than in the DO and solifenacin groups (1:0.34, Fig. 7a). This was confirmed using histological findings (Fig. 7b).

## Discussion

In our study, a mixture of berry extracts, the MO formula, was shown to be effective in controlling DO caused by BOO. We also observed a decrease in the level of the muscarinic receptor M2, and an increase in the level of M3. The levels of the pro-inflammatory cytokines and the index for the antioxidant effect were also observed to change due to the administration of the MO formula. Nitric oxide-related eNOS, Rock-I and -II, and Rho A could enhance the nitric oxide pathway including PDE5I. According to these results, the MO formula demonstrated antioxidant, anti-inflammatory, anti-muscarinic, and nitric oxide-enhancing effects.

Previous studies have presented that a mixture of berry extracts is related to the nitric oxide pathway, with the mixture exhibiting anti-inflammatory and antioxidant effects [6, 9, 16]. One study showed that the MO formula had lipid-regulating ability against dyslipidemia [7]. The mixture of berries consisted of *Lycium chinense Miller*, *Cornus officinalis Sieb, Robus coreanus Miquel, Cuscuta chinensis Lam* and *Schisandra chinense Baillon*. The marker compounds for the mixture were loganin, bertain, ellagic acid, hyperoside and schisandrin. *Lycium chinense Miller* functions as a protector from hepatotoxicity and nonspecific immune enhancement. *Cornus officinalis Sieb* is useful for bladder overactivity [17], erectile dysfunction [16], diabetes and diabetes-related chronic kidney disease [18]. *Cuscuta chinensis Lam* exhibits antioxidant [19] and anti-fibrotic effects [20]. *Robus coreanus Miquel* exhibits anti-inflammatory effects [21], is useful in erectile dysfunction [22] and increases sperm quality [23]. Lastly, the *Schisandra chinense Baillon* affects eNOS [24]. Based on the above information, the mixture of berry extracts results in multiple effects and actions. In Kampo medicine [5], a type of berry is used to control the symptoms related to the lower urinary tract. It is noteworthy that approximately 10 types of herbal medicine formulas are generally used in the urology department [5].

Many studies have demonstrated the effects of the nitric oxide pathway on symptoms related to lower urinary tract disorders [10, 11, 13, 14, 25–27]. In the study performed by Kim et al. [10], four different reasons were proposed to explain how the nitric oxide pathway was related to lower urinary tract symptoms/BPH. In BPH, the reduction in nitric oxide bioavailability was related to BPH and LUTS. In patients with BPH, eNOS and nNOS expression decreased in the prostate tissue compared to that in patients without BPH. These effects may be related to smooth muscle contraction in the bladder neck and prostate, which led to an increase in the bladder outlet resistance. In metabolic syndrome, the autonomic nervous system is overactivated by hyperinsulinemia. This may have been a result of the dysregulation of sympathetic and parasympathetic nerve tone. Increased sympathetic tone may lead to contraction of the smooth muscle in the prostate. The Rho kinase/Rho A pathway is also related to smooth muscle contraction of the bladder and induces changes in the compliance of the bladder and LUTS. In our study, eNOS and nNOS played an important role in the nitric oxide pathway, and previous studies have shown that the berry mixture affects the stimulation or enhancement of the nitric oxide pathway [6, 7, 9, 16]. In addition, there is evidence that the nitric oxide pathway and the related drug have an effect via controlling the ED and LUTS [10, 11, 13, 14, 25, 28]. Kedia et al. [25] observed that the enhancement of nitric oxide and the nitric oxide pathway signaling enzymes such as eNOS, nNOS, cGMP, cGK played an important role in smooth muscle contraction in human prostate. For this reason, PDE5I, a nitric oxide donor, was presented for the treatment of LUTS. Another study showed that the PDE5I played a role in enhancing the NO/cGMP signaling pathway which led to a relaxing of the human bladder, prostate and lower urinary tract vessel. These occurrences may increase the blood supply and oxygenation in these organs. PDE5I has also been shown to reduce non-voiding detrusor contraction and afferent nerve firing. Thus, in DO rats, PDE5I may be reduced by mechanosensitive afferent A- and C fibers [28].

Masuda et al. [29] observed that reactive oxygen species stimulated the capsaicin-sensitive C-fiber pathway and induced DO. Another study has reported that chronic BOO causes bladder wall over distention and leads the development of ischemia; the ischemia is a result of oxidative stress and was shown to induce LUTS [30]. For our animal model, we used a DO model induced by partial urethral obstruction. In approximately 2 weeks, urethral partial obstruction resulted in bladder overdistention and the distended bladder wall interfered with blood flow in the detrusor muscle. After the urethral obstruction was removed, the bladder was released from distention and blood flow restarted. However, following the re-initiation of blood flow, oxygen radical or oxidative stress occurred in the detrusor muscle. Thus, this may function as one of the causes of DO in our animal model. As identified in this study, an elevated level of SOD and a decreased level of 8-OHdG may function as a protective effect against this oxidative stress. Balachandran et al. [31] proposed that chronic urinary tract infection may be one of the causes of OAB/DO. Another study presented that covert infection may be the cause of the DO/OAB symptom [32]. The berry mixture formula improved the inflammation caused by cytokines and may also have a protective action against UTI and UTI-induced DO. Relative to oxidative stress, anti-inflammatory cytokines decreased the inflammation in BOO and may contribute to preserving the bladder function in the BOO model [33].

Polido et al. [34] demonstrated that intravesical oxybutynin had a protective effect on the functional and structural modifications of the detrusor muscle. The collagen concentration was lower in normal bladder and higher in the bladder of rats in the DO group. Like intravesical oxybutynin, the MO formula also had a similar effect on structural modification of the detrusor muscle. Based on the histological examination performed using a microscope, the muscle-collagen ratio was somewhat higher in the MO group than the control group; however, this ratio was significantly lower in the MO group compared to the solifenacin and DO groups. Thus, it could be deduced that the MO formula had a protective effect against structural modification while demonstrating a functional effect when compared to the other groups. Preservation of structural modification was also related to the antioxidant and anti-inflammatory effects of the MO formula.

The current study showed that the MO formula is effective against DO induced by partial urethral obstruction. Oxidative stress and inflammatory changes were reduced and structural modifications such as muscle-collagen ratio were also preserved. In addition, important molecular factors such as changes in the M3 and M2 receptors in OAB/DO were also preserved in the group administered the MO formula compared to those in the DO/solifenacin group. An enhanced nitric oxide pathway also contributed to the contraction interval and maximal detrusor pressure. Our study has several limitations. The MO formula was based on an oriental medicine consisting of a mixture of five types of berries. It was not revealed which specific material or molecule influenced the different effects observed in the experiment. There was also no evidence of cross-reaction or cross-acting. The precise molecule, mechanism, or pathway should be identified in a future study to find the exact activating substances from the five berries. This will demonstrate the direct cause of the antioxidant and anti-inflammatory effects observed through the modification of pro-inflammatory cytokines and antioxidant index materials. The preservation of the molecular change in the muscarinic receptor also warrants identification.

DO caused by partial urethral obstruction in rats was treated using a mixture of 5 berries (MO formula). By administering this formula, we observed an improvement in the function and structure of DO/OAB. These changes may be mediated by the nitric oxide pathway and the related anti-inflammatory and antioxidative effects exhibited by the berry mixture.

## Conclusions

The MO formula had similar pharmacologic effects to solifenacin. In addition, anti-inflammatory effect and antioxidant effects were observed when the MO formula was administered to rats. The enhancement of the nitric oxide pathway by the MO formula affected the DO induced by partial urethral obstruction in BPH-related DO. Thus, the berry mixture formula (MO formula) has been demonstrated as an option for the control of DO. Further studies are required to identify the exact molecule, mechanism, and active ingredient that cause the changes observed.

## Abbreviations

8-OHdG: 8-Hydroxy-2′-DeoxyGuanosine
BOO: Bladder Outlet Obstruction
BPH: Benign Prostate Hyperplasia
CMG: Cystometrogram
DO: Detrusor Overactivity
eNOS: endothelial Nitric Oxide Synthase
GMP: Guanylate Monophosphate
HPLC: High-performance liquid chromatography
MO formula: Modified Ojayeonjonghwan
NIH: National Institutes of Health
OAB: Overactive Bladder
PDE5I: Phosphodiesterase type 5 inhibitor
TNF-a: Tissue Necrosis Factor – a
